# Slowing the Progression of Diabetic Kidney Disease

**DOI:** 10.3390/cells12151975

**Published:** 2023-07-31

**Authors:** Olivia Blazek, George L. Bakris

**Affiliations:** Department of Medicine, American Heart Association Comprehensive Hypertension Center, The University of Chicago Medicine, Chicago, IL 60637, USA; olivia.blazek18@gmail.com

**Keywords:** kidney, nephropathy, diabetes, dialysis, guidelines

## Abstract

Diabetes is the most frequent cause of kidney disease that progresses to end-stage renal disease worldwide, and diabetic kidney disease is significantly related to unfavorable cardiovascular outcomes. Since the 1990s, specific therapies have emerged and been approved to slow the progression of diabetic kidney disease, namely, renin–angiotensin–aldosterone system blockers (including angiotensin-converting enzyme inhibitors (ACEi) angiotensin receptor blockers (ARBs), the non-steroidal mineralocorticoid receptor antagonist (NS-MRA), finerenone, and sodium–glucose cotransporter-2 (SGLT2) inhibitors). Mechanistically, these different classes of agents bring different anti-inflammatory, anti-fibrotic, and complementary hemodynamic effects to patients with diabetic kidney disease such that they have additive benefits on slowing disease progression. Within the coming year, there will be data on renal outcomes using the glucagon-like peptide-1 receptor agonist, semaglutide. All the aforementioned medications have also been shown to improve cardiovascular outcomes. Thus, all three classes (maximally dosed ACEi or ARB, low-dose SGLT-2 inhibitors, and the NS-MRA, finerenone) form the “pillars of therapy” such that, when used together, they maximally slow diabetic kidney disease progression. Ongoing studies aim to expand these pillars with additional medications to potentially normalize the decline in kidney function and reduce associated cardiovascular mortality.

## 1. Introduction

**Figure 1:**
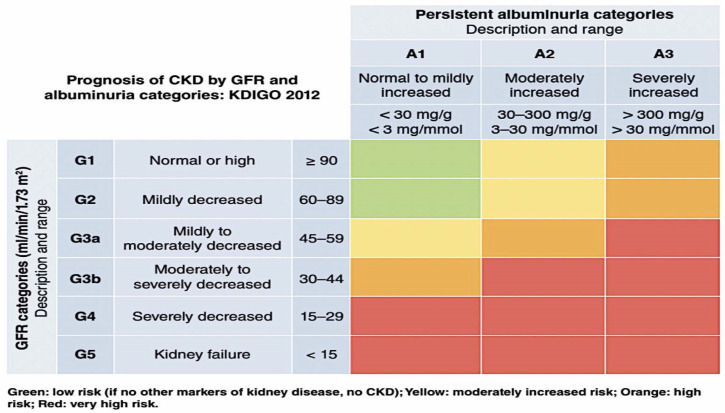
KDIGO heat map detailing the prognosis of CKD based upon GFR and albuminuria category [7]. Abbreviations: Kidney Disease: Improving Global Outcomes (KDIGO); GFR—glomerular filtration rate.

Chronic kidney disease (CKD) is characterized by a consistently diminished estimated glomerular filtration rate (eGFR) of less than 60 mL/min per 1.73 m^2^ with or without albuminuria (urine albumin to creatinine ratio [UACR] of less than 30 mg/g) for at least three months [1]. Approximately forty percent of people who have type 2 diabetes have a more advanced form of kidney disease as a result of their condition [2]. Diabetes-related kidney disease (DKD) is the most frequent cause of end-stage kidney disease (ESKD) in the world and is highly connected with unfavorable outcomes related to cardiovascular disease (CV) [3]. Because of the high incidence of morbidity and mortality associated with DKD, there is a significant amount of interest in reducing the development of the disease and decreasing the elevated risk of cardiovascular complications. The staging of kidney disease by the Kidney Disease Improving Global Outcomes (KDIGO) organization is shown in Figure 1. This helps identify the stage of disease for a particular person.

**Figure 2:**
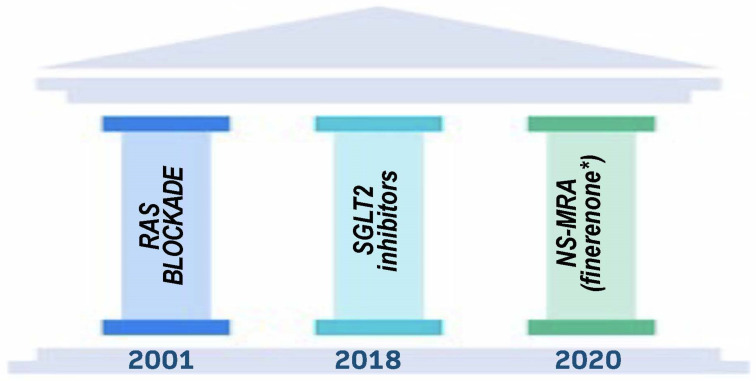
Pillars of Therapy to Reduce Cardio-Renal Risk by Year Approved. Note: (*) finerenone is singled out because only currently approved NS-MRA all others not currently involved or planned for trials dealing with cardio-renal outcomes.

There were no treatments available to reduce the progression of kidney damage linked with diabetes until the early 1990s. Despite this, a significant amount of fundamental research conducted in the 1980s, primarily by groups led by Brenner and Remuzzi, led to the discovery that the renin–angiotensin system is a key system that is overactive and contributes to a variety of intra-renal hemodynamic and cellular changes, as well as an ultimate decline in kidney function [4]. In animal models of type 1 diabetes, angiotensin-converting enzyme inhibitors (ACEIs) were shown to have a considerable advantage in avoiding morphologic alterations inside the kidney and preserving function [5]. This, in turn, translated into the Captopril trial being conducted on patients with type 1 diabetes and advanced kidney disease [6]. The results of this trial showed a significant slowdown in the course of kidney disease, which paved the way for a new age of kidney disease intervention [6]. In this article, we will discuss the class of medications known as renin–angiotensin–aldosterone system (RAAS) inhibitors that include angiotensin-converting enzyme inhibitors (ACEi) angiotensin receptor blockers (ARBs), as well as other more recent discoveries that help form the “pillars of therapy” to maximally slow nephropathy progression when used together in diabetic patients (Figure 2). This review will concentrate on individuals who have diabetes mellitus type 2 (T2DM), and it will also provide a quick overview of some of the mechanisms that are responsible for the therapeutic benefit.

## 2. Perspectives on Pathophysiology

One of the most important risk factors for the development of nephropathy over time is a persistent rise in glucose that is over the normal range. In addition to this, the prevalent accompanying condition of intraglomerular hypertension accelerates the decline in renal function even further [8]. It is important to note that microvascular complications, such as kidney disease, do not develop if the hemoglobin A1c level is kept below 6.5% [9]. Elevated glucose concentrations affect various cells in the kidney, including endothelial and mesangial cells as well as tubular cells [10,11,12]. High intracellular glucose concentrations coupled with increased insulin levels activate multiple metabolic and inflammatory pathways in these cells and result in the generation of toxic intermediates, advancing the progression of the disease.

Early manifestations of diabetic nephropathy include significant increases in glomerular filtration rate, mesangial expansion, and tubular and glomerular hypertrophy [12,13,14]. Additional early changes seen in the kidney are hypertrophy of the proximal tubule resulting from a high filtered glucose load. It is important to note that in animal models of DKD, tubular hypertrophy comes before glomerular hypertrophy. Both drugs that inhibit SGLT-2 and those that impede the renin–angiotensin–aldosterone system (RAAS) pathway can modify these pathophysiologic responses [8,14].

SGLT 2 inhibitors by preventing increased reabsorption of glucose and sodium chloride by the proximal tubules result in decreased sodium delivery to the macula densa cells of the juxtaglomerular apparatus (JGA). A mechanism that is related to decreased delivery of sodium to the JGA leads to (a) intrarenal activation of the renin–angiotensin–aldosterone system (RAAS) cascade, that subsequently results in efferent arteriolar vasoconstriction, increased intraglomerular pressure, and glomerular hyperfiltration, and (b) inhibition of adenosine production, which leads to afferent vasodilation and subsequent increased renal plasma flow. Persistent increases in intraglomerular pressure potentiate the increase in mechanical stress and increase oxygen demand, resulting in glomerular injury and the development and progression of kidney disease [8,14].

SGLT2 inhibitors have strong anti-inflammatory effects as a result of their ability to lower the nucleotide-binding domain, leucine-rich-containing family, and pyrin domain-containing-3 (NLRP3) protein. NLRP3 is the protein that is responsible for endothelial damage and increased fibrosis [15]. Of note, in animal models of DKD, tubular hypertrophy precedes glomerular hypertrophy, and inhibition of the tubular hypertrophy with an inhibitor of ornithine decarboxylase (the rate-limiting enzyme of polyamine synthesis) prevents glomerulosclerosis and development of DKD [16]. SGLT2 inhibitors also reduce a myriad of other proinflammatory factors and may contribute to promoting autophagy [17].

The NLRP3 inflammasome is a NOD-, LRR-, and pyrin domain-containing protein 3 and serves as an intracellular sensor that detects a broad range of microbial motifs, endogenous danger signals, and environmental irritants. When the cell detects these adverse responses, it results in the formation and activation of the NLRP3 inflammasome. Assembly of the NLRP3 inflammasome leads to caspase 1-dependent release of the pro-inflammatory cytokines IL-1β and IL-18, as well as to gasdermin D-mediated pyroptotic cell death [18].

SGLT2 inhibitors have been shown in several earlier investigations to reduce the activity of inflammatory indicators and to modulate the production of the NLRP3 protein. Empagliflozin may attenuate obesity-induced inflammatory responses by limiting macrophage activity and lowering TNF-a expression, as was proposed by some investigators [19]. In addition, research suggests that inhibiting SGLT-2 modifies the expression and activity of the NLRP3 inflammasome, which in turn has a favorable effect on renal function in mice with type 2 diabetes mellitus [20]. When SGLT2 inhibitors were provided, the same group showed diabetes-associated increases in inflammation-suppressed NLRP3 inflammasome activity as well as lower mRNA levels of ASC, IL-6, IL-1b, TNF-a, and caspase-1. This was the case regardless of whether or not glucose levels were reduced. Therefore, SGLT2 inhibitors are demonstrably anti-inflammatory drugs regardless of whether or not they can reduce glucose levels.

**Figure 3:**
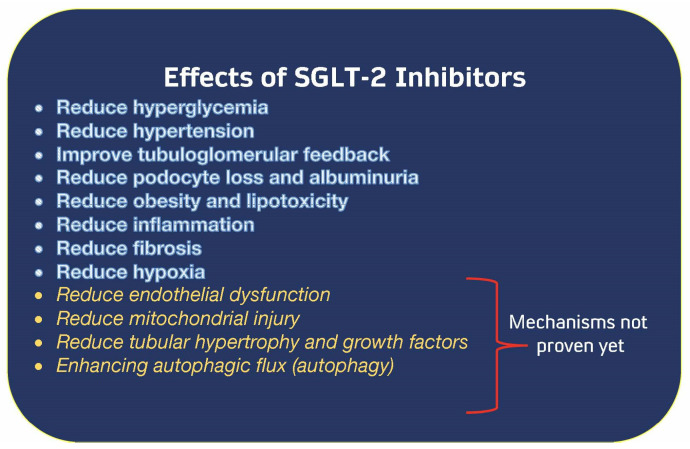
Known Mechanisms of SGLT-2 inhibitor action [14]. Abbreviations: SGLT-2—sodium–glucose cotransporter 2.

The most common consequences of type 2 diabetes are hypertension and damage to the kidneys. Expression of renal proximal tubular angiotensinogen (AGT) is affected by hyperglycemia, which stimulates AGT expression via enhanced oxidative stress. This in turn influences the development of hypertension and diabetic nephropathy. T2DM New Zealand obese mice fed a high-fat diet were used to test a hypothesis that SGLT2 inhibition prevents intrarenal AGT elevation and ameliorates kidney damage and hypertension in T2DM. Mice receiving a standard fat diet served as the control group. When DM mice showed signs of >350 milligrams per deciliter of blood glucose, both DM- and ND-fed mice were given 10 milligrams per kilogram per day of an SGLT2 inhibitor or a vehicle for a period of six weeks. The SGLT2 inhibitor was successful in normalizing the systolic blood pressure of diabetic mice, in addition to the hyperglycemia-induced rise in renal angiotensinogen mRNA and urine 8-isoprostane levels. The SGLT2 inhibitor was able to prevent the development of tubular fibrosis in diabetic mice. In addition, the SGLT2 inhibitor decreased the enhanced macrophage infiltration and cell proliferation that was linked with diabetes in the kidneys of diabetic mice. As a result, SGLT2 inhibitors cause alterations in the intra-renal renin–angiotensin system that are complementary to those brought about by RAS blockers [21]. A discussion of the mechanisms of the SGLT2 inhibitor class is beyond the scope of this review but are summarized in Figure 3.

Another hormone that, when produced in excessive quantities, leads to inflammation and fibrosis in the vascular tree, including the heart and the kidney [22], is called aldosterone. It is beyond the scope of this work to provide an in-depth analysis of the biology of aldosterone; nevertheless, the reader is directed to a recent review [23] for further information on this topic.

**Figure 4:**
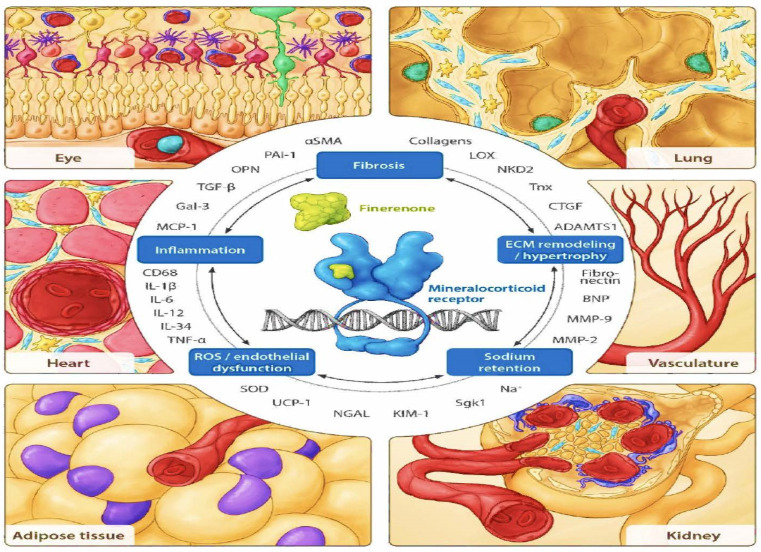
Components of pathophysiological MR overactivation counteracted by finerenone in different organs and cell types [28]. Organs including critical functional units (e.g., the retina in the eye, alveolus in the lung, and glomerulus in the kidney) and relevant specific cell types (e.g., fibroblasts in light blue and macrophages in yellow) with a documented MR-based pathophysiology, as described in the text, are framing the view. αSMA, alpha smooth muscle actin; ADAMTS1, a disintegrin and metalloproteinase with thrombospondin type 1 motif 1; BNP, B-type natriuretic peptide; CD68, cluster of differentiation 68; CTGF, connective tissue growth factor; ECM, extracellular matrix; Gal-3, galectin-3; IL, interleukin; KIM-1, kidney injury molecule 1; LOX, lysyl oxidase; MCP-1, monocyte chemoattractant protein-1; MMP, matrix metalloproteinase; NGAL, neutrophil gelatinase-associated lipocalin; NKD2, naked cuticle homolog 2; OPN, osteopontin; PAI-1, plasminogen activator inhibitor-1; ROS, reactive oxygen species; Sgk1, serum- and glucocorticoid-regulated kinase 1; SOD, superoxide dismutase; TGF-β, transforming growth factor-β; TNF-α, tumor necrosis factor-α; Tnx, tenascin-X; UCP-1, uncoupling protein-1.

Several studies suggest that the mineralocorticoid receptor is expressed in kidney cells other than the aldosterone-sensitive distal nephron. These kidney cells include vascular cells, podocytes, fibroblasts, and inflammatory cells [22,24] (Figure 4). These cells do not always co-express corticosteroid 11β-dehydrogenase isozyme 2 (11β-HSD2) with the mineralocorticoid receptor, which allows additional activation of the mineralocorticoid receptor by cortisol; 11β-HSD2 converts cortisol into its inactive metabolite cortisone [25]. In cases of chronic kidney disease and diabetes, aldosterone can be produced intrarenally, along with the local production of all other components of the RAAS. These other components include angiotensinogen at the proximal tubules [26,27] and renin release by the macula densa, as seen with hyperglycemia-induced mitochondrial succinate production acting on the GPR91 receptor.

Ectopic production of aldosterone can also occur in endothelium and smooth muscle cells, in neurons and glial cells, in adipocytes [26] and other types of cells. In cases of obesity that are associated with diabetes, there is a correlation between the production of aldosterone by adipocytes (also through aldosterone synthase) and vascular dysfunction caused by MR-mediated actions [29]. The traditional, genetic, and MR-mediated action of aldosterone on the distal tubules and collecting duct is to upregulate and increase the number of epithelial sodium channels present on the apical membrane. This occurs in response to the hormone’s effect on the collecting duct. These channels can also be found on the endothelium glycocalyx, vascular smooth muscle, the choroid plexus, and immune cells, and they react to vasopressin, angiotensin II, Rac1 [30] and insulin.

Cortisol binding to the mineralocorticoid receptor has been hypothesized to contribute to the overactivation and harmful impact of the mineralocorticoid receptor in cells in which 11 beta-HSD2 is neither expressed nor activated. This hypothesis has been put up as a possible explanation for these findings. In addition, aldosterone is not only secreted by the adrenal glands but also from the visceral adipocytes, which is especially the case in obese persons. It has been demonstrated that it contributes to inflammation and kidney injury, which is reflected by increases in albuminuria [31]. Evidence from a large, multicenter, prospective study of over one thousand newly diagnosed hypertensive patients that focused on the relationship between body mass index, aldosterone, plasma renin activity, and aldosterone-renin ratio [32]. Clear elevations of aldosterone were noted, with a stronger association in patients who were overweight and obese. Note that individuals diagnosed with primary aldosteronism did not show a correlation between the production of aldosterone and fat deposition, even though this linkage was discovered in patients with secondary aldosteronism and the bulk of the research that links the two together [33,34].

In addition to generating changes in gene expression, aldosterone has also been shown to have non-genomic effects on the epithelium and non-epithelial tissues [35]. These rapid effects include the production of reactive oxygen species (ROS) by NADPH oxidase, signaling through extracellular signal-regulated kinase 1 (ERK1), ERK2, and JUN (a component of the AP1 transcription factor), increased intracellular calcium, protein kinase C activation, and changes in pH [22]. It is important to note that the inhibition of these non-genomic mineralocorticoid-mediated effects of aldosterone.

In patients with diabetes, there is an activation of Rac-1, in addition to the aldosterone that is present. GTPases belong to the Rho family, and Rac-1 is a member of that family. It is an intracellular transducer that is recognized for its ability to regulate numerous signaling pathways, which are known to govern the structure of the cytoskeleton, transcription, and cell proliferation.

Rac-1 is an intracellular transducer known to regulate multiple signaling pathways that control cytoskeleton organization, transcription, and cell proliferation. There are several mechanisms that activate Rac1 by GTP loading including aldosterone, angiotensin II, TGF-β, high glucose, and interleukin (IL)-6/8 stimulation [23]. In diabetic nephropathy, high glucose levels activate the Rho pathway in mesangial cells. Rac1 appears to be essential for insulin signaling in muscle glucose uptake, which implicates Rac1 in human insulin resistance and dysglycemia [36]. In experimental models of diabetes, pharmacological inhibition of Rac1 attenuates endothelial dysfunction and also reduces platelet hyperaggregation in patients with diabetes [37]. In addition, glucose-induced Rac1 stimulation had a role in the activation of renal MR in cultured mesangial cells, which ultimately led to the death of those cells and nephropathy in an animal model of obesity-related type 2 diabetes (T2D) [38]. Increased levels of urine Rac1 were noted in focal segmental glomerular sclerosis and DKD, thus, it has the potential to be a novel biomarker (and a target for therapy) [39].

## 3. Blocking the RAAS and Its Clinical Applications

As a result of their ability to lower the risk of CKD progression [1], ACEIs and ARBs, are now required to be administered to patients diagnosed with DKD. It has been demonstrated that these drugs can lower intraglomerular hypertension, avoid structural harm to the glomerulus and other intrarenal structures, and lower albuminuria levels. The primary mechanism by which all of this is accomplished is through attenuating the effects of angiotensin II [40]. RAAS blockers have been shown to boost insulin sensitivity, leading to a reduction in insulin resistance as well as associated inflammation [41].

In 1993, a significant renal outcome study was conducted in patients with type 1 diabetes [6]. In 2001, two distinct clinical outcome studies in patients with DKD were published to evaluate the efficacy of two different ARBs [42,43]. In all three studies, there was a reduction in the advancement of nephropathy and cardiovascular events; however, the RENAAL study with losartan [43] showed the most substantial reduction in the progression of nephropathy leading to the need for dialysis. Both ACEi and ARBs substantially decreased the probability of developing ESKD by 13% and reduced the risk of serum creatinine doubling by 29% [44], according to a meta-analysis of randomized controlled studies that compared the effects of different antihypertensive medicines on the development of DKD.

An established indication of inflammation is a moderately increased level of albumin in the urine, which was originally referred to as microalbuminuria but is now called moderately increased albuminuria. This condition was first described in this context in 2014 [45]. Patients with diabetes, regardless of the type, benefit from a reduction in albuminuria of up to 30–40% when they take ACEi or ARBs. This is more than twice as much as any other antihypertensive class that has been used. As a result, variations in blood pressure are not the only possible explanation for these observations. In point of fact, alterations in glomerular permeability and selectivity contribute to this impact, and this has been observed with diltiazem, a calcium channel blocker that is not dihydropyridine types [8,46]. The Food and Drug Administration considers a reduction in albuminuria of more than 30 percent that is maintained over a period of time to be a surrogate sign of delaying the course of chronic kidney disease [47] because these data, when combined with an examination of data from more recent clinical trials, have led to this conclusion.

When administered in the early stages of the illness, maximum tolerable dosages of an ACEi or an ARB can delay the development of albuminuria and lower any amounts that may already be present. The BENEDICT trial was the first large-scale prospective investigation to indicate that the longest-acting ACEi, trandolapril, might prevent the start of albuminuria in persons with type 2 diabetes irrespective of blood pressure reduction when compared to placebo or the calcium channel blocker, verapamil [48]. This finding was made possible by the fact that the BENEDICT trial was the first study of its kind. Telmisartan or enalapril was given to each of the 250 participants in a different research project that was prospective, multicenter, double-blind, and lasted for five years. The participants all had type 2 diabetes and early nephropathy. The primary endpoint was a change in the glomerular filtration rate at five years, which was evaluated by the plasma clearance of iohexol. Alterations in the amount of albumin that was excreted in the urine, cardiovascular events, and death from any cause were secondary objectives. There was no discernible difference between the groups in the primary endpoint. In addition, the study demonstrated that telmisartan was not inferior to enalapril from a statistical standpoint. In addition, there was no change in the secondary endpoints that were measured [49].

Following these studies, there were uncertainties regarding the extent of RAAS inhibition and the possibility that combination treatment with two RAAS blockers may have synergistic effects on DKD. Three separate studies sought to demonstrate superior results, but all of them were unsuccessful [50,51,52,53]. In addition, the combination groups experienced the greatest incidences of hyperkalemia across all of these studies.

RAAS blockers at the highest tolerated dose continue to be the first-line treatment for decreasing the course of DKD; nevertheless, these medications are unable to completely stop the disease process [1,3,54]. The effectiveness of these drugs can be affected by a variety of personal circumstances, including variations in genetic makeup, the amount of sodium consumed in the diet, an intolerance to the prescription, and the administration of a dose that is less than the recommended maximum, a dose used in trials. In addition to just blocking RAAS in part, these classes do not address several additional pathways involved in the pathogenesis of DKD. However, novel classes of drugs have been developed to augment the effects of RAAS blockage. Nevertheless, RAAS blockers are the first line of defense in the fight against the advancement of nephropathy.

## 4. Mineralocorticoid Receptor Antagonists: Nonsteroidal (NS-MRA) and Steroidal (S-MRA)

Nonsteroidal (NS-MRA) and steroidal (S-MRA) mineralocorticoid receptor antagonists are both types of mineralocorticoid receptor antagonists. The “escape” or “breakthrough” of aldosterone [55] is one of the probable reasons why RAAS inhibitors do not provide the full advantages that they could. This is a common occurrence in patients who are being treated for an extended period with RAAS blockers, and it is associated with a faster drop in eGFR as well as increases in albuminuria in DKD patients over time [55,56].

Aldosterone produces various alterations in the immune system, increases oxidative stress, and eventually vascular injury [22] when it persistently activates the mineralocorticoid receptor. If aldosterone stimulation continues, these and other alterations will lead to inflammatory and fibrotic changes in the kidney, heart, and vasculature, all of which will contribute to the continuous advancement of glomerulosclerosis and the increase in albuminuria [13,57,58]. The use of mineralocorticoid receptor antagonists (MRAs), which are based on the consequences of MR activation, has the potential to ultimately assist in slowing the course of DKD and minimize the risk of heart failure. In spite of the fact that the MR receptor is produced in every kidney cell, people with diabetes have a higher level of receptor expression in particular kidney cells in comparison to healthy controls [59].

Heart failure is the cardiovascular consequence that occurs most frequently when diabetic nephropathy progresses to a more advanced stage [60]. There have been two heart failure outcome trials with S-MRAs, and both of them indicate a clear mortality decrease field [61,62]. Both of these trials used S-MRAs. However, because persons with DKD tend to have substantial hyperkalemia risk, there are no studies done on renal outcomes using S-MRA drugs.

A novel class of agents known as NS-MRA have a lower risk of hyperkalemia and finerenone is the only one of five in the class that has been testing both heart failure and kidney disease outcomes [63]. Specifically, finerenone is the only NS-MRA that has demonstrated a delayed progression of nephropathy and a reduction in heart failure hospitalization in patients who have diabetes and various levels of kidney disease [64,65,66].

Two distinct studies were conducted, each of which followed the same protocol but used different inclusion criteria but recruitment locations were otherwise comparable. Both of these studies looked at patients who had type 2 diabetes and varying degrees of kidney function (CKD stages 2–4), Figure 3. The primary objective of each study was distinct from the others: FIDELIO-DKD measured renal function, whereas FIGARO-DKD focused on cardiovascular outcomes [67,68]. When completed, the data on each patient were compiled into a single database, and the subsequent analysis of more than 13,000 individuals resulted in the FIDELITY analysis [66].

According to the findings of the FIDELITY analysis, there was a substantial decrease of 20% in the progression to dialysis and a reduction of 22% in heart failure hospitalizations. Furthermore, an on-treatment analysis of the all-cause mortality outcomes in the FIDELITY trial showed that the group of patients on finerenone had a reduced incidence of all-cause mortality (HR 0.82, 95% CI 0.70–0.96, *p* = 0.014) and cardiovascular mortality (HR 0.82, 95% CI 0.67–0.99, *p* = 0.040) in comparison to the placebo group. This was the case across a wide range of CKD stages and baseline UACR values. In the group that was intended to receive treatment, finerenone was shown to reduce the incidence of sudden cardiac death (hazard ratio = 0.75, 95% confidence interval = 0.57–0.996, *p* = 0.046) [69].

When it comes to the danger of hyperkalemia in advanced kidney disease, NS-MRAs appear to be safer than their steroidal cousins. A comparison using individuals with the same level of kidney function, and blood pressure with diabetes revealed that the incidence of hyperkalemia was only one-sixth as common [70,71]. In addition, the use of finerenone in conjunction with an SGLT-2 inhibitor can lessen the likelihood of developing hyperkalemia as a side effect of finerenone [70].

It is unknown what mechanism accounts for the difference in the impact that NS-MRAs and S-MRAs have on potassium handling; nevertheless, a similar phenomenon has been seen with Ocedureone, a distinct NS-MRA that is being developed for resistant hypertension and which has a half-life of 52 h compared to finerenone’s half-life of 2–3 h [72]. As a result, the reasoning about the variances in spironolactone’s half-life cannot be used to adequately explain the discrepancies.

## 5. Sodium–Glucose Co-Transporter 2 (SGLT-2) Inhibitors

This family of medicines diminishes hyperglycemia when kidney function is good; however, when they were put through clinical trials, they were found to protect kidney function and reduce the number of hospitalizations for heart failure despite having no effect on glucose levels [73]. In addition, the methods by which these pharmaceuticals work fall into at least 11 distinct categories; hence, no one mechanism can be specifically attributed to the positive effects of these medications [17,74,75,76]. The known mechanisms of SGLT-2 inhibition are summarized in (Figure 3) [14].

When taken with RAAS blockers, this family of drugs has been shown to improve renal outcomes in individuals both with and without diabetes [73,77]. More specifically, this class of pharmaceuticals has been shown to reduce the progression to ESKD and the requirement for dialysis in these patients. This improvement was constant independent of the degree of albuminuria or cardiovascular illness that existed at the beginning of the study.

SGLT-2 inhibitors have been given the green light for usage in individuals who have an eGFR that is as low as 20 mL/min/1.73 m^2^ [1]. The use of SGLT-2 inhibitors was linked with a 29% reduction in risk of cardiovascular events (HR 0.71, 95% CI 0.63–0.80) [78] in a meta-analysis of individuals with DKD stage 3–4. The study was conducted on patients in the advanced stages of the disease. Data on their use in patients with a GFR of 30 mL/min per 1.73 m^2^ come from a subgroup analysis of the CREDENCE trial. This trial demonstrated that canagliflozin slowed the progression of kidney disease without an increase in kidney-related adverse events or acute kidney injury when compared to patients with an eGFR of 30 mL/min per 1.73 m^2^ [79]. At similar lower levels of eGFR, data from subgroups of a separate study with a different SGLT2 inhibitor also revealed kidney and heart benefit [80]. In addition, a comprehensive meta-analysis of SGLT-2 inhibitors indicated a lower risk of cardiovascular mortality (HR 0.85, 95% CI 0.78–0.93), heart failure hospitalization (HR 0.68, 95% CI 0.61–0.76), and major adverse cardiovascular events (HR 90, 95% CI 0.85–0.95) [77]. These findings were based on a reduction in the risk of cardiovascular death, heart failure hospitalization, and major adverse cardiovascular events.

People with or without diabetes who suffer from albuminuric renal disease and/or heart failure are now required to take SGLT2 inhibitors as part of their therapy regimen. This pharmacological class is eligible for consideration as an extra third pillar to assist in the slowing down of the course of renal disease.

## 6. Utilization of “Pillar Therapy” in the Treatment of DKD

In all of the trials, the renal and cardiovascular results of utilizing either SGLT2 inhibitors or finerenone on background RAAS blockade were examined, and as was previously noted, the outcomes were favorable. Nevertheless, the use of all three agents is restricted. A post hoc examination of the data from the finerenone trials involving participants who were also receiving SGLT2 inhibitors [81] have produced some interesting findings. In this subgroup study of patients who were receiving concurrent treatment with finerenone and an SGLT-2 inhibitor, the cardiorenal advantages of finerenone were found to continue independent of the use of the SGLT-2 inhibitor. In addition to this, there was a considerable additional drop in the number of hospitalizations for heart failure in the group that was getting all three medication classes [81].

## 7. GLP-1 RA Stands for Glucagon-like Peptide-1 Receptor Agonists

A recent meta-analysis of approximately 60,000 patients with T2DM treated with GLP-1 RAs had a significant reduction in the composite kidney outcome (development of macroalbuminuria, doubling of serum creatinine, 40% or greater decline in eGFR, kidney replacement therapy, or death from kidney disease) when compared to placebo (HR 0.79, 95% CI 0.673–0.87) as well as a trend towards a reduction in worsening kidney function (HR 0.86, 95% CI 0.72–1.02) [82].

It is not known which mechanism(s) or processes GLP-1 RAs use to delay the course of DKD; however, recent research that has not yet been published shows immunological pathways may be involved. These findings are hypothesis-generating [83,84]; nevertheless, post hoc analysis of studies using liraglutide and semaglutide does indicate substantial decreases in albuminuria and a delayed drop in eGFR. There is currently one trial that is being conducted to explicitly assess the renal effects of the use of the GLP-1 RA, semaglutide, in persons who have advanced DKD. This trial is called the FLOW [85], and it will test the effect of semaglutide on the development of DKD. The findings of this trial should hopefully be available by the end of 2024.

The FIDELITY analysis investigated and assessed the usage of GLP-1 RAs in conjunction with finerenone and RAAS blocking. An exploratory subgroup analysis was conducted to assess the impact that the usage of GLP-1 RA has on the therapeutic effect of finerenone. At the time of the study, 394 (or 6.9%) of the 5674 patients being evaluated were receiving GLP-1 RAs. There was no correlation between baseline GLP-1 RA usage and the extent of UACR decrease achieved with finerenone in the study [86]. There was also not a discernible difference in renal outcomes based on the usage of GLP-1 RA at baseline (the *p*-value for the interaction was 0.15 and the *p*-value for the main effect was 0.51) [86].

## 8. Conclusions

Building upon the concept used by cardiologists in heart failure, for the first time, nephrologists and endocrinologists can apply the principles of three established “pillars of therapy”—ACEi or ARB (at maximally tolerated doses), SGLT2 inhibitors, and finerenone (NS-MRA)—to maximally slow declines in kidney function, as shown in Figure 4. When the FLOW trial is finished in 2024, perhaps there may be four pillars of therapy to use. Future ongoing studies will assess renal outcomes in patients with DKD using these medications in combination.

## Data Availability

Not applicable.

## References

[1] de Boer I.H., Khunti K., Sadusky T., Tuttle K.R., Neumiller J.J., Rhee C.M., Rosas S.E., Rossing P., Bakris G. (2022). Diabetes Management in Chronic Kidney Disease: A Consensus Report by the American Diabetes Association (ADA) and Kidney Disease: Improving Global Outcomes (KDIGO). Diabetes Care.

[2] Fried L.F., Folkerts K., Smeta B., Bowrin K.D., Mernagh P., Millier A., Kovesdy C.P. (2021). Targeted literature review of the burden of illness in patients with chronic kidney disease and type 2 diabetes. Am. J. Manag. Care.

[3] DeFronzo R.A., Bakris G.L. (2022). Modifying chronic kidney disease progression with the mineralocorticoid receptor antagonist finerenone in patients with type 2 diabetes. Diabetes Obes. Metab..

[4] Bhandari S., Mehta S., Khwaja A., Cleland J.G., Ives N., Brettell E., Chadburn M., Cockwell P. (2022). Renin–Angiotensin System Inhibition in Advanced Chronic Kidney Disease. N. Engl. J. Med..

[5] Anderson S., Brenner B.M. (1988). Intraglomerular Hypertension: Implications and Drug Treatment. Annu. Rev. Med..

[6] Lewis E.J., Hunsicker L.G., Bain R.P., Rohde R.D. (1993). The Effect of Angiotensin-Converting-Enzyme Inhibition on Diabetic Nephropathy. N. Engl. J. Med..

[7] Andrassy K.M. (2013). Comments on ‘KDIGO 2012 clinical practice guideline for the evaluation and management of chronic kidney disease’. Kidney Int..

[8] Brown S.A., Walton C.L., Crawford P., Bakris G.L. (1993). Long-term effects of antihypertensive regimens on renal hemodynamics and proteinuria. Kidney Int..

[9] International Expert Committee (2009). International Expert Committee report on the role of the A1C assay in the diagnosis of diabetes. Diabetes Care.

[10] Thomas M.C., Brownlee M., Susztak K., Sharma K., Jandeleit-Dahm K.A.M., Zoungas S., Rossing P., Groop P.-H., Cooper M.E. (2015). Diabetic kidney disease. Nat. Rev. Dis. Prim..

[11] Bakris G.L., Fairbanks R., Traish A.M. (1991). Arginine vasopressin stimulates human mesangial cell production of endothelin. J. Clin. Investig..

[12] Gaber L., Walton C., Brown S., Bakris G. (1994). Effects of different antihypertensive treatments on morphologic progression of diabetic nephropathy in uninephrectomized dogs. Kidney Int..

[13] Barrera-Chimal J., Bonnard B., Jaisser F. (2022). Roles of Mineralocorticoid Receptors in Cardiovascular and Cardiorenal Diseases. Annu. Rev. Physiol..

[14] DeFronzo R.A., Reeves W.B., Awad A.S. (2021). Pathophysiology of diabetic kidney disease: Impact of SGLT2 inhibitors. Nat. Rev. Nephrol..

[15] Yaribeygi H., Butler A.E., Atkin S.L., Katsiki N., Sahebkar A. (2018). Sodium–glucose cotransporter 2 inhibitors and inflammation in chronic kidney disease: Possible molecular pathways. J. Cell. Physiol..

[16] Thomson S.C., Deng A., Bao D., Satriano J., Blantz R.C., Vallon V. (2001). Ornithine decarboxylase, kidney size, and the tubular hypothesis of glomerular hyperfiltration in experimental diabetes. J. Clin. Investig..

[17] Packer M. (2022). Critical Reanalysis of the Mechanisms Underlying the Cardiorenal Benefits of SGLT2 Inhibitors and Reaffirmation of the Nutrient Deprivation Signaling/Autophagy Hypothesis. Circulation.

[18] Yaribeygi H., Katsiki N., Butler A.E., Sahebkar A. (2019). Effects of antidiabetic drugs on NLRP3 inflammasome activity, with a focus on diabetic kidneys. Drug Discov. Today.

[19] Xu L., Nagata N., Nagashimada M., Zhuge F., Ni Y., Chen G., Mayoux E., Kaneko S., Ota T. (2017). SGLT2 Inhibition by Empagliflozin Promotes Fat Utilization and Browning and Attenuates Inflammation and Insulin Resistance by Polarizing M2 Macrophages in Diet-induced Obese Mice. Ebiomedicine.

[20] Birnbaum Y., Bajaj M., Yang H.-C., Ye Y. (2018). Combined SGLT2 and DPP4 Inhibition Reduces the Activation of the Nlrp3/ASC Inflammasome and Attenuates the Development of Diabetic Nephropathy in Mice with Type 2 Diabetes. Cardiovasc. Drugs Ther..

[21] Woods T.C., Satou R., Miyata K., Katsurada A., Dugas C.M., Klingenberg N.C., Fonseca V.A., Navar L.G. (2019). Canagliflozin Prevents Intrarenal Angiotensinogen Augmentation and Mitigates Kidney Injury and Hypertension in Mouse Model of Type 2 Diabetes Mellitus. Am. J. Nephrol..

[22] Funder J.W. (2017). Aldosterone and Mineralocorticoid Receptors—Physiology and Pathophysiology. Int. J. Mol. Sci..

[23] Mende C.W., Samarakoon R., Higgins P.J. (2023). Mineralocorticoid Receptor-Associated Mechanisms in Diabetic Kidney Disease and Clinical Significance of Mineralocorticoid Receptor Antagonists. Am. J. Nephrol..

[24] Williams G. (2019). Aldosterone: The Missing Cardiorenal Link. Am. J. Nephrol..

[25] Gomez-Sanchez E.P., Gomez-Sanchez C.E. (2021). 11β-hydroxysteroid dehydrogenases: A growing multi-tasking family. Mol. Cell. Endocrinol..

[26] Peti-Peterdi J. (2010). High glucose and renin release: The role of succinate and GPR91. Kidney Int..

[27] Siragy H.M., Carey R.M. (2010). Role of the Intrarenal Renin-Angiotensin-Aldosterone System in Chronic Kidney Disease. Am. J. Nephrol..

[28] Kolkhof P., Lawatscheck R., Filippatos G., Bakris G.L. (2022). Nonsteroidal Mineralocorticoid Receptor Antagonism by Finerenone—Translational Aspects and Clinical Perspectives across Multiple Organ Systems. Int. J. Mol. Sci..

[29] Briones A.M., Cat A.N.D., Callera G.E., Yogi A., Burger D., He Y., Corrêa J.W., Gagnon A.M., Gomez-Sanchez C.E., Gomez-Sanchez E.P. (2012). Adipocytes Produce Aldosterone Through Calcineurin-Dependent Signaling Pathways. Hypertension.

[30] Pearce D., Soundararajan R., Trimpert C., Kashlan O.B., Deen P.M., Kohan D.E. (2015). Collecting Duct Principal Cell Transport Processes and Their Regulation. Clin. J. Am. Soc. Nephrol..

[31] Barrera-Chimal J., Lima-Posada I., Bakris G.L., Jaisser F. (2021). Mineralocorticoid receptor antagonists in diabetic kidney disease—Mechanistic and therapeutic effects. Nat. Rev. Nephrol..

[32] Rossi G.-P., Sechi L.A., Giacchetti G., Ronconi V., Strazzullo P., Funder J.W. (2008). Primary aldosteronism: Cardiovascular, renal and metabolic implications. Trends Endocrinol. Metab..

[33] Flynn C. (2014). Increased Aldosterone: Mechanism of Hypertension in Obesity. Semin. Nephrol..

[34] Flynn C., Bakris G.L. (2011). Interaction between Adiponectin and Aldosterone. Cardiorenal Med..

[35] Funder J.W. (2006). Aldosterone and the Cardiovascular System: Genomic and Nongenomic Effects. Endocrinology.

[36] Kawarazaki W., Nagase M., Yoshida S., Takeuchi M., Ishizawa K., Ayuzawa N., Ueda K., Fujita T. (2012). Angiotensin II- and Salt-Induced Kidney Injury through Rac1-Mediated Mineralocorticoid Receptor Activation. J. Am. Soc. Nephrol..

[37] Patel S., Tang J., Overstreet J.M., Anorga S., Lian F., Arnouk A., Goldschmeding R., Higgins P.J., Samarakoon R. (2019). Rac-GTPase promotes fibrotic TGF-β1 signaling and chronic kidney disease via EGFR, p53, and Hippo/YAP/TAZ pathways. FASEB J..

[38] Tung C.-W., Hsu Y.-C., Shih Y.-H., Chang P.-J., Lin C.-L. (2018). Glomerular mesangial cell and podocyte injuries in diabetic nephropathy. Nephrology.

[39] Saleem M.A., Welsh G.I. (2019). Podocyte RhoGTPases: New therapeutic targets for nephrotic syndrome?. F1000Research.

[40] Ruggenenti P., Cravedi P., Remuzzi G. (2010). The RAAS in the pathogenesis and treatment of diabetic nephropathy. Nat. Rev. Nephrol..

[41] Kuang Z., Hou N., Kan C., Han F., Qiu H., Sun X. (2022). The protective effects of SGLT-2 inhibitors, GLP-1 receptor agonists, and RAAS blockers against renal injury in patients with type 2 diabetes. Int. Urol. Nephrol..

[42] Lewis E.J., Hunsicker L.G., Clarke W.R., Berl T., Pohl M.A., Lewis J.B., Ritz E., Atkins R.C., Rohde R., Raz I. (2001). Renoprotective Effect of the Angiotensin-Receptor Antagonist Irbesartan in Patients with Nephropathy Due to Type 2 Diabetes. N. Engl. J. Med..

[43] Brenner B.M., Cooper M.E., De Zeeuw D., Keane W.F., Mitch W.E., Parving H.-H., Remuzzi G., Snapinn S.M., Zhang Z., Shahinfar S. (2001). Effects of Losartan on Renal and Cardiovascular Outcomes in Patients with Type 2 Diabetes and Nephropathy. N. Engl. J. Med..

[44] Casas J.P., Chua W., Loukogeorgakis S., Vallance P., Smeeth L., Hingorani A.D., MacAllister R.J. (2005). Effect of inhibitors of the renin-angiotensin system and other antihypertensive drugs on renal outcomes: Systematic review and meta-analysis. Lancet.

[45] Bakris G.L., Molitch M. (2014). Microalbuminuria as a Risk Predictor in Diabetes: The Continuing Saga. Diabetes Care.

[46] Smith A.C., Toto R., Bakris G.L. (1998). Differential effects of calcium channel blockers on size selectivity of proteinuria in diabetic glomerulopathy. Kidney Int..

[47] Levey A.S., Gansevoort R.T., Coresh J., Inker L.A., Heerspink H.L., Grams M.E., Greene T., Tighiouart H., Matsushita K., Ballew S.H. (2020). Change in Albuminuria and GFR as End Points for Clinical Trials in Early Stages of CKD: A Scientific Workshop Sponsored by the National Kidney Foundation in Collaboration With the US Food and Drug Administration and European Medicines Agency. Am. J. Kidney Dis..

[48] Ruggenenti P., Fassi A., Ilieva A.P., Bruno S., Iliev I.P., Brusegan V., Rubis N., Gherardi G., Arnoldi F., Ganeva M. (2004). Preventing Microalbuminuria in Type 2 Diabetes. N. Engl. J. Med..

[49] Barnett A.H., Bain S.C., Bouter P., Karlberg B., Madsbad S., Jervell J., Mustonen J. (2004). Angiotensin-Receptor Blockade versus Converting–Enzyme Inhibition in Type 2 Diabetes and Nephropathy. N. Engl. J. Med..

[50] Ruggenenti P., Cortinovis M., Parvanova A., Trillini M., Iliev I.P., Bossi A.C., Belviso A., Aparicio M.C., Trevisan R., Rota S. (2021). Preventing microalbuminuria with benazepril, valsartan, and benazepril–valsartan combination therapy in diabetic patients with high-normal albuminuria: A prospective, randomized, open-label, blinded endpoint (PROBE) study. PLoS Med..

[51] Fried L.F., Emanuele N., Zhang J.H., Brophy M., Conner T.A., Duckworth W., Leehey D.J., McCullough P.A., O’Connor T., Palevsky P.M. (2013). Combined Angiotensin Inhibition for the Treatment of Diabetic Nephropathy. N. Engl. J. Med..

[52] Parving H.-H., Brenner B.M., McMurray J.J., de Zeeuw D., Haffner S.M., Solomon S.D., Chaturvedi N., Persson F., Desai A.S., Nicolaides M. (2012). Cardiorenal End Points in a Trial of Aliskiren for Type 2 Diabetes. N. Engl. J. Med..

[53] Heagerty A., Yusuf S., Teo K.K., Pogue J., Dyal L., Copland I., Schumacher H., Dagenais G., Sleight P., Anderson C. (2008). Telmisartan, Ramipril, or Both in Patients at High Risk for Vascular Events. N. Engl. J. Med..

[54] Sarafidis P.A., Stafylas P.C., Kanaki A.I., Lasaridis A.N. (2008). Effects of Renin-Angiotensin System Blockers on Renal Outcomes and All-cause Mortality in Patients With Diabetic Nephropathy: An Updated Meta-analysis. Am. J. Hypertens..

[55] Bomback A.S., Klemmer P.J. (2007). The incidence and implications of aldosterone breakthrough. Nat. Clin. Pract. Nephrol..

[56] Sato A., Hayashi K., Naruse M., Saruta T. (2003). Effectiveness of Aldosterone Blockade in Patients With Diabetic Nephropathy. Hypertension.

[57] Bertocchio J.-P., Warnock D.G., Jaisser F. (2011). Mineralocorticoid receptor activation and blockade: An emerging paradigm in chronic kidney disease. Kidney Int..

[58] Agarwal R., Kolkhof P., Bakris G., Bauersachs J., Haller H., Wada T., Zannad F. (2020). Steroidal and non-steroidal mineralocorticoid receptor antagonists in cardiorenal medicine. Eur. Heart J..

[59] Wilson P.C., Wu H., Kirita Y., Uchimura K., Ledru N., Rennke H.G., Welling P.A., Waikar S.S., Humphreys B.D. (2019). The single-cell transcriptomic landscape of early human diabetic nephropathy. Proc. Natl. Acad. Sci. USA.

[60] House A.A. (2018). Management of Heart Failure in Advancing CKD: Core Curriculum 2018. Am. J. Kidney Dis..

[61] Pitt B., Zannad F., Remme W.J., Cody R., Castaigne A., Perez A., Palensky J., Wittes J. (1999). The effect of spironolactone on morbidity and mortality in patients with severe heart failure. Randomized Aldactone Evaluation Study Investigators. N. Engl. J. Med..

[62] Pitt B., Remme W., Zannad F., Neaton J., Martinez F., Roniker B., Bittman R., Hurley S., Kleiman J., Gatlin M. (2003). Eplerenone, a Selective Aldosterone Blocker, in Patients with Left Ventricular Dysfunction after Myocardial Infarction. N. Engl. J. Med..

[63] Kintscher U., Bakris G.L., Kolkhof P. (2022). Novel non-steroidal mineralocorticoid receptor antagonists in cardiorenal disease. Br. J. Pharmacol..

[64] Bakris G.L., Agarwal R., Anker S.D., Pitt B., Ruilope L.M., Rossing P., Kolkhof P., Nowack C., Schloemer P., Joseph A. (2020). Effect of Finerenone on Chronic Kidney Disease Outcomes in Type 2 Diabetes. N. Engl. J. Med..

[65] Pitt B., Filippatos G., Agarwal R., Anker S.D., Bakris G.L., Rossing P., Joseph A., Kolkhof P., Nowack C., Schloemer P. (2021). Cardiovascular Events with Finerenone in Kidney Disease and Type 2 Diabetes. N. Engl. J. Med..

[66] Agarwal R., Filippatos G., Pitt B., Anker S.D., Rossing P., Joseph A., Kolkhof P., Nowack C., Gebel M., Ruilope L.M. (2021). Cardiovascular and kidney outcomes with finerenone in patients with type 2 diabetes and chronic kidney disease: The FIDELITY pooled analysis. Eur. Heart J..

[67] Bakris G.L., Agarwal R., Anker S.D., Pitt B., Ruilope L.M., Nowack C., Kolkhof P., Ferreira A.C., Schloemer P., Filippatos G. (2019). Design and Baseline Characteristics of the Finerenone in Reducing Kidney Failure and Disease Progression in Diabetic Kidney Disease Trial. Am. J. Nephrol..

[68] Ruilope L.M., Agarwal R., Anker S.D., Bakris G.L., Filippatos G., Nowack C., Kolkhof P., Joseph A., Mentenich N., Pitt B. (2019). Design and Baseline Characteristics of the Finerenone in Reducing Cardiovascular Mortality and Morbidity in Diabetic Kidney Disease Trial. Am. J. Nephrol..

[69] Filippatos G., Anker S.D., August P., Coats A.J.S., Januzzi J.L., Mankovsky B., Rossing P., Ruilope L.M., Pitt B., Sarafidis P. (2023). Finerenone and effects on mortality in chronic kidney disease and type 2 diabetes: A FIDELITY analysis. Eur. Heart J.—Cardiovasc. Pharmacother..

[70] Agarwal R., Joseph A., Anker S.D., Filippatos G., Rossing P., Ruilope L.M., Pitt B., Kolkhof P., Scott C., Lawatscheck R. (2022). Hyperkalemia Risk with Finerenone: Results from the FIDELIO-DKD Trial. J. Am. Soc. Nephrol..

[71] Agarwal R., Pitt B., Palmer B.F., Kovesdy C.P., Burgess E., Filippatos G., Małyszko J., Ruilope L.M., Rossignol P., Rossing P. (2022). A comparative post hoc analysis of finerenone and spironolactone in resistant hypertension in moderate-to-advanced chronic kidney disease. Clin. Kidney J..

[72] Bakris G., Pergola P.E., Delgado B., Genov D., Doliashvili T., Vo N., Yang Y.F., McCabe J., Benn V., Pitt B. (2021). Effect of KBP-5074 on Blood Pressure in Advanced Chronic Kidney Disease: Results of the BLOCK-CKD Study. Hypertension.

[73] Ma Y., Lin C., Cai X., Hu S., Zhu X., Lv F., Yang W., Ji L. (2023). Baseline eGFR, albuminuria and renal outcomes in patients with SGLT2 inhibitor treatment: An updated meta-analysis. Acta Diabetol..

[74] Tuttle K.R. (2023). Digging deep into cells to find mechanisms of kidney protection by SGLT2 inhibitors. J. Clin. Investig..

[75] Packer M. (2023). Mechanisms of enhanced renal and hepatic erythropoietin synthesis by sodium–glucose cotransporter 2 inhibitors. Eur. Heart J..

[76] Theofilis P., Oikonomou E., Tsioufis K., Tousoulis D. (2023). Diabetes Mellitus and Heart Failure: Epidemiology, Pathophysiologic Mechanisms, and the Role of SGLT2 Inhibitors. Life.

[77] McGuire D.K., Shih W.J., Cosentino F., Charbonnel B., Cherney D.Z.I., Dagogo-Jack S., Pratley R., Greenberg M., Wang S., Huyck S. (2021). Association of SGLT2 Inhibitors With Cardiovascular and Kidney Outcomes in Patients With Type 2 Diabetes. JAMA Cardiol..

[78] Li N., Zhou G., Zheng Y., Lv D., Zhu X., Wei P., Zheng M., Liu S., Zhou E., Sun W. (2022). Effects of SGLT2 inhibitors on cardiovascular outcomes in patients with stage 3/4 CKD: A meta-analysis. PLoS ONE.

[79] Bakris G., Oshima M., Mahaffey K.W., Agarwal R., Cannon C.P., Capuano G., Charytan D.M., De Zeeuw D., Edwards R., Greene T. (2020). Effects of Canagliflozin in Patients with Baseline eGFR < 30 mL/min per 1.73 m^2^. Clin. J. Am. Soc. Nephrol..

[80] Heerspink H.J.L., Jongs N., Chertow G.M., Langkilde A.M., McMurray J.J.V., Correa-Rotter R., Rossing P., Sjöström C.D., Stefansson B.V., Toto R.D. (2021). Effect of dapagliflozin on the rate of decline in kidney function in patients with chronic kidney disease with and without type 2 diabetes: A prespecified analysis from the DAPA-CKD trial. Lancet Diabetes Endocrinol..

[81] Rossing P., Anker S.D., Filippatos G., Pitt B., Ruilope L.M., Birkenfeld A.L., McGill J.B., Rosas S.E., Joseph A., Gebel M. (2022). Finerenone in Patients With Chronic Kidney Disease and Type 2 Diabetes by Sodium–Glucose Cotransporter 2 Inhibitor Treatment: The FIDELITY Analysis. Diabetes Care.

[82] Sattar N., Lee M.M.Y., Kristensen S.L., Branch K.R.H., Del Prato S., Khurmi N.S., Lam C.S.P., Lopes R.D., McMurray J.J.V., Pratley R.E. (2021). Cardiovascular, mortality, and kidney outcomes with GLP-1 receptor agonists in patients with type 2 diabetes: A systematic review and meta-analysis of randomised trials. Lancet Diabetes Endocrinol..

[83] Tonneijck L., Van Raalte D.H., Muskiet M.H.A. (2017). Liraglutide and Renal Outcomes in Type 2 Diabetes. N. Engl. J. Med..

[84] Marso S.P., Bain S.C., Consoli A., Eliaschewitz F.G., Jódar E., Leiter L.A., Lingvay I., Rosenstock J., Seufert J., Warren M.L. (2016). Semaglutide and Cardiovascular Outcomes in Patients with Type 2 Diabetes. N. Engl. J. Med..

[85] Rossing P., Baeres F.M.M., Bakris G., Bosch-Traberg H., Gislum M., Gough S.C.L., Idorn T., Lawson J., Mahaffey K.W., Mann J.F.E. (2023). The rationale, design and baseline data of FLOW, a kidney outcomes trial with once-weekly semaglutide in people with type 2 diabetes and chronic kidney disease. Nephrol. Dial. Transplant..

[86] Rossing P., Agarwal R., Anker S.D., Filippatos G., Pitt B., Ruilope L.M., Amod A., Marre M., Joseph A., Lage A. (2021). Efficacy and safety of finerenone in patients with chronic kidney disease and type 2 diabetes by GLP-1RA treatment: A subgroup analysis from the FIDELIO-DKD trial. Diabetes Obes. Metab..

